# Dynamic lighting mitigates photoperiodic injury in greenhouse tomatoes

**DOI:** 10.3389/fpls.2026.1731972

**Published:** 2026-02-04

**Authors:** Jason Lanoue, Sarah St. Louis, Celeste Little, Saman Soltaninejad, Rose Seguin, Xiuming Hao

**Affiliations:** 1 Harrow Research and Development Centre, Agriculture & Agri-Food Canada (AAFC), Harrow, ON, Canada; 2 Sollum Technologies Inc., Montréal, QC, Canada

**Keywords:** CEA (controlled environment agriculture), circadian rhythm, continuous light, dynamic light, greenhouse, LED (light-emitting diode), light spectrum, photoperiod

## Abstract

The light-limiting winter months in high latitude countries pose problems for producers trying to produce fresh fruit and vegetables year-round. Supplemental electric lighting is usually required. However, utilizing electric lighting, even high-efficacy LED fixtures, results in high electricity costs. Long photoperiod of low intensity lighting (up to 24 h (hours) is a promising strategy to meet plants’ light requirement [daily light integral (DLI)], which can be implemented in many countries as utility companies incentivize the use of low-cost, off-peak electricity use. In this study, we compared a conventional 16 h white light treatment (Control) to a 24 h treatment which involved a change from white light during the day to blue light at night at a reduced photosynthetic photon flux density (PPFD; dynamic) and a static light treatment which kept both spectrum and PPFD constant for 24 h on 2 cherry tomato cultivars. In addition, each treatment also had a low blue (10%) and high blue (30%) variation. All treatments had the same DLI. It was found that the 24 h dynamic lighting strategies had similar maximum quantum yield of photosystem II (F_v_/F_m_) as the 16 h controls while that at 24 h static treatments were drastically reduced. In addition, the F_v_/F_m_ value from the 24 h static treatment with high blue content was the lowest among all treatments indicating high blue light may be detrimental during a static 24 h photoperiod. In addition, the overall yield from the 24 h dynamic treatments were similar to the 16 h conventional treatments while the 24 h static treatments were significantly lower. Taken together, these results indicated that a 24 h dynamic light treatment is an effective strategy to mitigate photoperiodic injury and the light recipe with low blue light is more energy-efficient. Compared to the control 16 h photoperiod, a 24 h dynamic lighting strategy can reduce electricity costs due to lower nighttime prices, electricity monthly delivery charge and capital cost, due to reduced peak light intensity and fixture installation while maintaining fruit yield and quality in greenhouse cherry production.

## Introduction

Tomatoes are one of the most popular fruits globally due to their versatility and high nutrient profile. In high latitude regions which experience cold, dark winters, consumers are reliant on imports for fresh tomatoes. However, there has been a growing trend over the last few decades where off-season tomato production in these countries is met, or at least augmented, by greenhouse production. In 2023, the Canadian greenhouse market produced over 314, 000 metric tons of tomato, accounting for nearly 40% of all greenhouse production and approximately $870 million in whole-sell value (AAFC, 2025). While greenhouses allow for the sheltering of crops from harsh conditions during the winter months, by themselves, they do little to improve the poor light environment during these periods. In Harrow, Ontario, Canada, where this experiment takes place (30 minutes away from the second highest concentration of high-tech greenhouses in the world), the daily light integral (DLI) during the winter months is typically between 5–15 mol m^-2^ d^-1^ (Korczynski et al., 2002; Faust and Logan, 2018). With traditional cover materials (i.e., glass and double-poly) and shading from greenhouse structure, the typical DLI within the greenhouse may be half of that outside (Roberts, 1998). With optimal DLI for tomato production being between 20–30 mol m^-2^ d^-1^ (OMAFRA, 2025), the light environment within the greenhouse during the winter months in northern high latitude countries is sub-optimal.

Electric lighting systems such as light-emitting diode (LED) fixtures can supplement the low solar radiation during the winter months to improve production. Typically implemented as overhead lighting with a photosynthetic photon flux density (PPFD) between 200-250 µmol m^-2^ s^-1^ and photoperiods around 16 h (hours), supplemental lighting has long proven to be essential for high yielding winter greenhouse tomato production (Mcavoy and Janes, 1984; Mcavoy et al., 1989; Appolloni et al., 2021; Heuvelink et al., 2024). However, utilizing electric lighting, even high efficacy LEDs, comes with a trade-off – electricity costs can be high. In some regions of the world, the utilization of LED fixtures to supplement the low natural solar radiation can account for up to 30% of the growers operating costs (Jayalath et al., 2024).

A recent trend in greenhouse production of all fruits and vegetables has been to reduce costs while also increasing sustainability through the use of energy sources which emit lower greenhouse gas emissions (GHG) (Ashton et al., 2024). However, while trying to reduce costs and GHGs it is important to, at minimum, maintain yield and fruit quality. With respect to lighting, in order to maintain yield specifically, target/desired DLI must be maintained.

Although it may seem counter intuitive, photoperiodic extension while maintaining DLI can reduce electricity costs (both electricity itself and the monthly delivery charge (based on the electricity usage in the 1 peak hour in a month (IESO, 2025), in addition to light fixture costs) and preferentially utilize electricity which is created from low CO_2_ emitting sources during the night. In Ontario, Canada, baseload electricity comes mainly from nuclear and hydro sources, both with low GHG emissions (NREL, 2021). During peak demand hours, standby natural gas generators supplement supply, but they emit significant CO_2_. To discourage peak-time usage, utilities employ time-of-use pricing (IESO, 2025). Popular in regions of North America (NYSERDA, 2025) and Europe (IRENA, 2019), time-of-use pricing systems offers low electricity prices during periods of low demand such as during the night. Algorithms such as DynaGrow have shown promise in locations like Denmark where preferentially using lighting during periods of low electricity cost showed potential savings of 25-50% (Sorensen et al., 2016). While traditional industries such as manufacturing lack the flexibility to alter electricity use pattern, greenhouse production can take advantage of this time-of-use electricity pricing.

Photoperiodic extension beyond the conventional 16 h period, up to and including 24 h (continuous light; CL), can allow for more freedom in the gamification (selectively using the electricity during low-cost periods) of electricity grids, empowering growers to realize cost savings while also mitigating GHG emissions. In this way, using an extended photoperiod can preferentially utilize cheaper, off-peak electricity. While photoperiodic extension up to 24 h lighting has been theorized to increase yield (Velez-Ramirez et al., 2012), this has not yet come to fruition. CL (24 h) lighting is known to cause interveinal chlorosis, characterized by yellowing of the leaves thus reducing photosynthetic capacity and ultimately yield (Velez-Ramirez et al., 2011). The most popular hypothesis for why plants are unable to grow under 24 h lighting is an imbalance in gene expression, specifically those involved in light capture. The free-running circadian cycle within a plant is highly important in regulating key biochemical and physiological responses. When this is interrupted by environmental factors, such as CL, key gene expression can be disturbed, ultimately leading to leaf injury; such is the case with *chlorophyll a/b binding protein* (*CAB)* in photoperiodic injury (Velez-Ramirez et al., 2014).

However, some studies have shown that environmental variations such as temperature between the day and subjective night period (i.e., the period which would dark during a traditional 16 h photoperiod) under 24 h lighting may help mitigate injury. For example, the introduction of a thermoperiod with large differences between day and night temperature are initiated have shown reduced CL injury (Haque et al., 2017). For this reason, we aimed to understand whether a dynamic (changing PPFD and spectrum) or static (constant PPFD and spectrum) 24 h light treatment had an impact on overall tomato plant health compared to a conventional 16 h control. It was hypothesized that utilizing a dynamic 24 h light treatment would reduce or even eliminate photoperiodic injury in greenhouse tomato compared to a 16 h photoperiod resulting in similar yield between photoperiods. In addition we hypothesized that the use of a dynamic 24 h photoperiod can eliminate photoperiod injury compared to a static 24 h lighting strategy where injury would occur.

Under a continuous photoperiod, plants are exposed to constant light. Being photoautotrophs, plant utilize light to create the sugar building blocks needed for growth. Because of this, plants do not have a mechanism which allows them to turn off their light capturing ability. Under 24 h lighting, an increase in photo-oxidative stress via the increase in reactive oxygen species (ROS) has been observed which can cause DNA damage and be harmful to the plant (Pulido et al., 2010; Huang et al., 2019; Liu and Liu, 2024). Blue light can have a profound influence on regulating the photo-oxidative state in plants (Chibani et al., 2025). However, in tomato, there is a trade off between the amount of blue light and overall yield – too much blue light can cause a decrease in yield (Kaiser et al., 2018). We hypothesized that an increase in blue light during CL could aid in mitigating photoperiodic damage potentially due to the influence on antioxidant capacity. Therefore, we further examine the impact of low blue (10%) and high blue (30%) light spectrum on tomato plant production.

## Materials and methods

### Plant material and experimental design

Seeds of cherry tomato (*Solanum lycopersicum*) cv. ‘Tomary’ and ‘Black Cherry’ were sown into rockwool plugs on October 12^th^, 2022. On October 27^th^, 2022, seedlings were transplanted into rockwool (10x10 cm; Grodania A/S, Milton, Ontario, Canada) plugs and placed into a glass greenhouse at Harrow Research and Development Centre (Agriculture & Agri-Food Canada, Harrow, Ontario, Canada; 42.03°N, 82.90°W). Plants were exposed to 150 µmol m^-2^ s^-1^ of high-pressure sodium lighting for 16 hours per day with a temperature between 22-25°C during the day and 19 ± 0.5°C at night. On November 24^th^, 2022, the seedlings at 4 true leaf stage were placed on 50cm Grodan Prestige (50x15x10 cm) slabs in a 200 m^2^ glass greenhouse at the Harrow Research and Development Centre. Plants were exposed to 200 µmol m^-2^ s^-1^ of broad spectrum white light for 16 h. Single stemmed plants were used resulting in a stem density of 5 stems m^-2^. The plants were drip-irrigated with a complete nutrient solutions with an EC of 3.0 dS m^-1^ and pH 5.8, respectively (OMAFRA, 2010). The average daytime temperature was held between 22-25°C depending on ambient solar radiation while the nighttime temperature was 19 ± 0.5°C. The relative humidity was 70 ± 10% during both the day and nighttime periods. The greenhouse was enriched to a CO_2_ level of 1000 µL L^-1^ when not vented.

**Figure 1:**
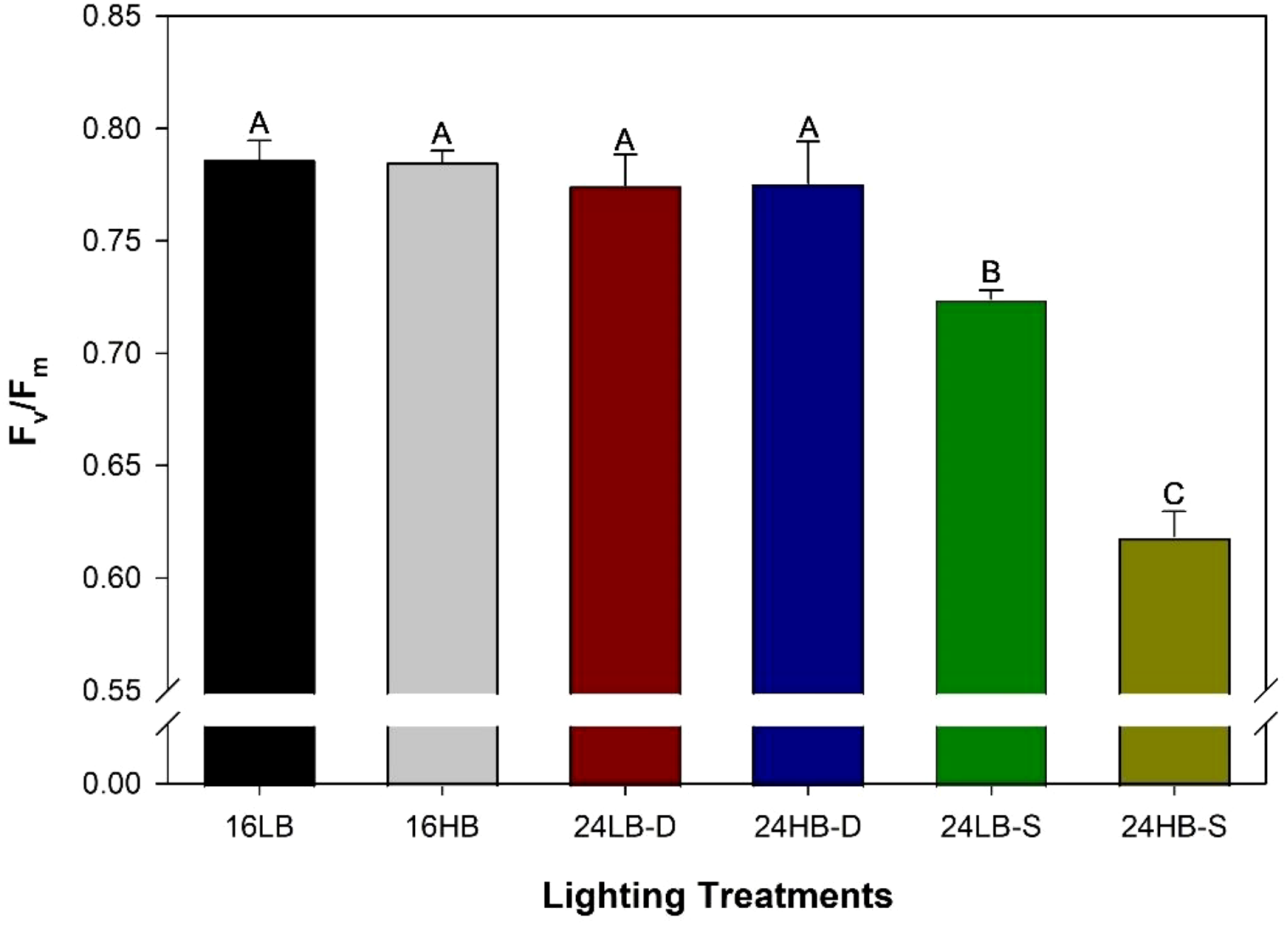
Dark-adapted maximum efficiency of PSII (F_v_/F_m_) from the fifth leaf from the apex of cv. ‘Tomary’ grown under various lighting treatments as determined between February 6^th^-10^th^, 2023. The data represents the average and standard error of the mean (n=3) of three randomly selected leaves for each treatment. Letter groups (A–C) represent significant difference between the lighting treatments at p<0.05 with a *post-hoc* Tukey-Kramer adjustment.

The greenhouse was subdivided into a completely randomized design containing 18 plots measuring 3mx1.6m with 24 total plants (12 of each cultivar) within. On December 6^th^, 2022, six supplemental overhead light treatments were randomly assigned to the plots (**Table 1**). All light treatments were replicated three times. The light was provided via Sollum SF05B multi-channel LED lighting fixtures (Sollum Technologies Inc. Montréal, Quebéc, Canada). Spectral composition was determined using a Li-COR spectroradiometer (Li-180, Li-COR Biosciences Inc., Lincoln, NE, USA) at the apex of the plant during the night to exclude any solar radiation (**Figure 1**; **Supplementary Figure 1**). The PPFD was also measured during the night period at 2 locations within each plot at the top of the plant with a one meter quantum line sensor (Li-COR 191R; Li-COR Biosciences Inc., Lincoln, NE, USA). Light abatement curtains ran the length of the compartment to stop contamination between light treatments. The curtains were opened on sunny days to minimize shading and were closed during cloudy days and during the night period. The daily light integral (DLI) from the supplemental light in all treatments was approximately 14.4 mol m^-2^ d^-1^ (**Table 1**) Lights remained on regardless of natural solar radiation level in order to ensure the same total DLI (solar + supplemental) was given to the plants in all treatments. The 24LB-S and 24HB-S have the lowest monthly electricity delivery charge because they have the lowest peak light intensity (peak hour electricity usage).

**Table 1:** Overhead supplemental light treatments (16LB – 16 h low blue, 16HB – 16 h high blue, 24LB-D – 24 h low blue dynamic lighting, 24HB-D – 24 h high blue dynamic lighting, 24LB-S – 24 h low blue static lighting, 24HB-S – 24 h high blue static lighting) provided by Sollum SF05B LED light fixtures.

Treatment	Photoperiod (h)	Duration (hh:mm)	Spectrum (R:G:B)	PPFD (µmol m^-2^ s^-1^)	Daily Light Integral (DLI, mol m^-2^ s^-1^)
16LB	16	6:00-22:00	62:28:10	250 ± 4	14.4
16HB	16	6:00-22:00	42:28:30	250 ± 3	14.4
24LB-D	16	6:00-22:00	62:28:10	212 ± 1	12.2
	8	22:00-6:00	0:0:100	75 ± 2	2.2
24HB-D	16	6:00-22:00	42:28:30	212 ± 1	12.2
	8	22:00-6:00	0:0:100	75 ± 2	2.2
24LB-S	24	6:00-6:00	62:28:10	168 ± 4	14.5
24HB-S	24	6:00-6:00	42:28:30	167 ± 3	14.4

PPFD measurements were done using a Li-COR 191R one-meter quantum line sensor at two locations within each plot at the apex of the crop canopy. Red was defined as wavelength between 600-700nm, green was wavelengths between 500-599nm, and blue was wavelengths between 400-499nm. Grey areas indicate nighttime light intensity and spectrum.

### Morphological measurements

On March 22^nd^, 2023, morphological measurements were performed on 6 randomly selected plants from each cultivar grown under each light treatment (2 plants from each replication). The internode length was determined by measuring from the apex of the plant to the 10^th^ node. This distance was then divided by the number of nodes to get the internode distance. The stem diameter was measured using a digital caliper between the 4^th^ and 5^th^ node. Leaf length and width of the 5^th^ leaf was measured using a ruler measuring from the tip of the leaf to the base connecting to the stem and at the widest point of the leaflets. SPAD values, a measure of leaf greenness often correlated with chlorophyll content, was measured by taking the average of 5 measurements per leaf from each treatment (SPAD Model 502, Konica Minolta, Osaka, Japan).

### Leaf gas exchange and chlorophyll fluorescence

Leaf gas exchange and chlorophyll fluorescence measurements were focused on cv. ‘Tomary’ due to its relevance to the greenhouse industry. Measurements began on February 6^th^, 2023, and culminated on February 10^th^, 2023. One leaf located at the fifth node on three separate plants were randomly selected from each lighting treatment and was wrapped in aluminum foil to dark-adapt for 20 minutes. The leaf was then placed in a 2 cm^2^ chamber of a Li-COR 6800 (Li-COR Biosciences Inc., Lincoln, NE, USA) fluorometer head attachment. The leaf temperature was set to 22°C, relative humidity of 65%, and CO_2_ concentration of 1000 µL L^-1^. The minimum fluorescence in a dark-adapted state (F_o_) was collected after which an 800 ms saturation red light pulse (8000 µmol m^-2^ s^-1^) was emitted to the leaf to obtain the maximum fluorescence (F_m_). The variable fluorescence in a dark-adapted state (F_v_) was then calculated (F_v_=F_m_-F_o_) to determine the maximum quantum efficiency of photosystem II (PSII) in the dark-adapted state (F_v_/F_m_).

Next, the same leaf was exposed and acclimated to 1500 µmol m^-2^ s^-1^ of 90% red and 10% blue light until steady-state photosynthesis and fluorescence (F_t_) were achieved. Once stable, a saturating pulse was given to the leaf (F’_m_) followed by a far-red light pulse (25 µmol m^-2^ s^-1^; F’_o_). The light level was then reduced in a step-wise fashion down to 0 µmol m^-2^ s^-1^. At each light level, the photosynthetic rate and fluorescence were allowed to stabilize during a 10 minute period. The light-adapted efficiency of PSII photochemistry (φPSII=(F’_m_-F_t_)/F’_m_), electron transport rate (ETR=φPSII*PPFD*0.5), and non-photochemical quenching (NPQ=(F_m_-F’_m_/F’_m_)) were calculated for each light level. At the 0 µmol m^-2^ s^-1^ level, only the photosynthetic rate was measured.

### Yield

All cherry tomato clusters were pruned to have maximum of 14 fruits per cluster. Cherry tomato harvest began on January 24^th^, 2023 and continued until April 17^th^. Harvest occurred every fourth day with the exception of weekends and holidays once the fruit reached full size and 80% of the cluster fruits had changed colour. A colour change was deemed to occur when the ‘Tomary’ fruit were light red to red and ‘Black Cherry’ accumulated their distinctive purple colour.

### Electricity use, cost, and efficiency

The electricity used by each lighting treatment was obtained from Sollum Technologies SUNaaS^®^ platform. This platform allows for real time electricity usage for each treatment (accounting for dimming and spectrum). The total electricity consumed by each treatment was calculated by using the kW for each treatment and multiplying it by the number of hours each treatment was on throughout the production period.

Total electricity costs were calculated using the electricity consumed by each treatment and the hourly price throughout the production period (IESO, 2025). A summation of hourly electricity cost plus the monthly delivery charge [a flat rate of $9.15/kWh based on the electricity used during a peak hour in each month (OEB, 2022)] allowed for the total electricity cost per treatment.

The electricity-cost-efficiency (ECE; $ g^-1^ fruit weight) and electricity-use-efficiency (kWh g^-1^fruit weight) were calculated by dividing the cost of electricity and electricity used by the total fruit weight at the end of the experiment.

### Soluble solids content (°Brix)

On February 24^th^, fruits of both cv. ‘Tomary’ and ‘Black Cherry’ under all lighting treatments were harvested. The ripe fruits with full colour change were removed from their cluster and pooled. Six randomly selected fruits were then cut in half and carefully squeezed to extract juice from the flesh. The juice was placed on a Atago PR-101α digital refractometer (Atago Co. Ltd. Tokyo, Japan) for soluble solids content (i.e., °Brix) measurements. Due to time constraints, only six fruit from each cultivar were used for the soluble solid measurement and they were only conducted at one time point throughout the production period.

### Sample extraction for antioxidant analysis

On February 24^th^, fruits from both cv. ‘Tomary’ and ‘Black Cherry’ under all lighting treatments were harvested. The fruits from the pooled harvest were mixed and six randomly selected ripe fruit were used for the analysis. Due to time constraints, only 6 fruit from each cultivar were used for the following analysis. While this is a limitation of the study, it allowed for comparison between treatments. These fruit were different than the ones used in the soluble solids measurements. The fruits were cut in half and the placental tissue and seeds were removed. The remaining flesh and skin were placed in a 50 mL tube, flash frozen in liquid nitrogen and immediately placed in a -80°C freezer until further analysis. For extraction, tubes were removed from the -80°C freezer and placed in a lyophilizer (FreeZone 4.5L -84°C; Labconco, Kansas City, MO, USA) for 72h. Once lyophilized, stainless steel beads were added to the 50mL tube and the samples were homogenized (Bead Ruptor Elite; Omni International, Kennesaw, GA, USA). The homogenized tissue was sub-sampled into 1.7mL microfuge tubes to which 1mL of 99% methanol was added. The microfuge tube was placed on a nutator for 24h. The sample was centrifuged at 15, 000 rpm for 10 minutes to allow for extraction of the supernatant which was placed in a clean 15mL tube. An additional 1mL of fresh 99% methanol was added to the centrifuge tube which was vortexed then placed on a nutator for an additional 6h after which the sample was again centrifuged and the supernatant was added to the subsequent sample (2mL of total methanolic sample). Methanolic sample extracts were kept in a -20°C freezer until needed.

#### Ferric reducing antioxidant power assay

The ferric reducing antioxidant power (FRAP) assay measurements were conducted in a similar manner as previously described (Lanoue et al., 2022). FRAP reagent was made immediately before the analysis and consisted of 300mM acetate buffer (pH 3.6), 20mM FeCl_2_, and 10mM 2, 4, 6- Tris(2-pyridyl)-s-triazine (TPTZ). 25µL of methanolic sample extract and 75µL of 99% methanol were added to a microfuge tube followed by 900µL of FRAP reagent. The microfuge tube was then placed in a heating block at 37°C for 1h. The content of the microfuge tube was then transferred to a polystyrene micro-cuvette (path length = 1cm) and the absorbance was measured at 593nm. A standard curve was completed utilizing the same experimental protocol but with ascorbic acid in place of the sample.

#### 2, 2-diphenyl-1-picrylhydrazyl assay

DPPH reagent (350µM) was freshly prepared prior to the beginning of the assay and kept in the dark. 125µL of methanolic extract and 1000µL of DPPH reagents were added to a polystyrene cuvette and mixed. The solution was then incubated at room temperature in the dark for 30 minutes before the absorbance was measured at 517nm. A standard curve was completed using the same experimental protocol by with ascorbic acid in place of the sample.

#### Anthocyanin content

Determination of anthocyanin content was done using a modified protocol from (Lee et al., 2005). 100µL of methanolic extract was added to both 1mL of potassium chloride (0.025M; pH = 1.0) and 1mL of sodium acetate (0.4M; pH = 4.5) in separate 15mL tubes and incubated at room temperature for 30 minutes. The mixtures were then placed in separate polystyrene cuvettes and absorbance for both was measured at 520nm and 700nm. The anthocyanin content was calculated using the following equation:


$$Anthocyanin\;Content\;\left(mg\;C3G\;g^{-1}\;DW\right):\frac{\left(A*MW*DF*10^{3}\right)}{\epsilon *l}$$

Where *A* is the absorbance (*A*=(*A*_520nm_-*A*_700nm_)_pH1.0_ – (*A*_520nm_-*A*_700nm_)_pH4.5_), *MW* is the molecular weight of cyanidin-3-glucoside (449.2 g mol^-1^), *DF* was the dilution factor, 10^3^ is the factor to convert from g to mg, *ϵ* is the molar extinction coefficient of cyanidin-3-glucoside (26, 900 L mol^-1^), and *l* is the path length of the cuvette (1 cm).

#### Phenolic content

The phenolic content was determined using a modified protocol from (Ainsworth and Gillespie, 2007). 100µL of methanolic extract, 200µL of Folin-Ciocalteu’s phenol reagent (2N; Millipore Sigma Supelco; Oakville, ON, Canada), and 800µL of 700mM sodium carbonate were added to a microfuge tube. The microfuge tube was vortexed for 30 seconds than incubated at room temperature for 2h. The solution was placed in a polystyrene cuvette and the absorbance was measured at 765nm. A standard curve was completed using the same experimental protocol but with gallic acid in place of the sample.

### Statistical analysis

The experiment was conducted in a completely randomized design with each treatment having 3 replicates. All statistics were performed using SAS Studio 3.5. After the analysis of variance (one-way ANOVA), a multiple means comparison between the different lighting treatments was done using a Tukey-Kramer adjustment and a value of p<0.05 indicates a significant difference.

## Results

### Plant growth

The internode length and stem diameter as well as leaf length and width of ‘Tomary’ were unaffected by lighting treatments (**Table 2**). In addition, the SPAD measurement, often correlated with chlorophyll content was similar among all light treatments in ‘Tomary’. In effect, neither the addition of blue light (i.e., HB), nor the extension to photoperiod (i.e., 24h) impacted the morphological properties in this cultivar.

**Table 2:** Morphological responses of cv ‘Tomary’ and ‘Black Cherry’ to various overhead supplemental light treatments as determined on March 22nd, 2023.

Treatment	Internode length (cm)	Stem diameter (mm)	5^th^ leaf length (cm)	5^th^ leaf width (cm)	SPAD of 5^th^ leaf
Tomary					
16LB	5.7 ± 0.1^A^	7.2 ± 0.3^A^	31.5 ± 0.8^A^	20.3 ± 0.3^A^	44.5 ± 1.1^A^
16HB	5.7 ± 0.2^A^	7.1 ± 0.6^A^	34.0 ± 0.7^A^	23.7 ± 1.3^A^	44.5 ± 2.0^A^
24LB-D	5.7 ± 0.3^A^	7.3 ± 0.6^A^	31.8 ± 0.9^A^	21.0 ± 1.2^A^	49.1 ± 2.4^A^
24HB-D	5.3 ± 0.2^A^	7.1 ± 0.4^A^	31.7 ± 1.1^A^	21.7 ± 1.0^A^	48.2 ± 1.1^A^
24LB-S	6.0 ± 0.3^A^	8.4 ± 0.3^A^	31.8 ± 1.2^A^	21.4 ± 1.5^A^	47.4 ± 0.5^A^
24HB-S	6.1 ± 0.1^A^	8.0 ± 0.3^A^	31.0 ± 1.4^A^	22.0 ± 1.8^A^	47.7 ± 1.3^A^
Black Cherry					
16LB	7.3 ± 0.1^b^	7.5 ± 0.5^b^	29.7 ± 1.1^a^	20.0 ± 1.8^a^	42.2 ± 1.5^a^
16HB	7.4 ± 0.1^b^	9.1 ± 0.8^ab^	32.2 ± 1.1^a^	21.2 ± 1.5^a^	43.2 ± 2.3^a^
24LB-D	8.3 ± 0.2^a^	7.3 ± 0.6^b^	28.8 ± 1.3^a^	19.7 ± 1.5^a^	42.3 ± 1.4^a^
24HB-D	8.4 ± 0.2^a^	8.7 ± 0.4^ab^	32.2 ± 1.6^a^	25.0 ± 2.1^a^	39.7 ± 1.9^a^
24LB-S	8.2 ± 0.2^a^	10.1 ± 0.7^a^	31.3 ± 0.9^a^	24.0 ± 1.6^a^	41.6 ± 0.9^a^
24HB-S	8.6 ± 2.9^a^	9.7 ± 0.5^ab^	30.9 ± 0.5^a^	25.6 ± 1.8^a^	41.4 ± 1.6^a^

The data represents the average and standard error of 6 randomly selected plants per cultivar. Different letter groups (A, B, C) represent a statistical difference as determine by a one-way ANOVA with a Tukey-Kramer adjustment (p<0.05) with a *post-hoc* Tukey-Kramer adjustment. Upper case letters signify statistical differences for ‘Tomary’ and lower case letters for ‘Black Cherry’.

In ‘Black Cherry’, plants grown under 16h of lighting regardless of blue light content were observed to have the shortest internodes (**Table 2**). With respect to stem diameter, plants grown under 24LB-S had the thickest stem while those grown under both 16LB and 24LB-D had the thinnest stem. Similar to the results in ‘Tomary’, the leaf length, width, and SPAD measurement in ‘Black Cherry’ were similar under all lighting treatments.

### Leaf chlorophyll fluorescence and photosynthesis

Dark-adapted chlorophyll fluorescence measurement provides valuable insight into the plant’s light harvesting complex, therefore, revealing the plants’ ability to absorb light (Baker, 2008). The maximum efficiency of photosystem II (PSII; F_v_/F_m_) is a common metric used to assess plant stress. In our experiment, plants grown under the 16LB, 16HB, 24LB-D, and 24HB-D had similarly high values of F_v_/F_m_ which indicate the plant is in an unstressed or low stress environment allowing for light capture and absorption to occur readily (**Figure 1**). However, both 24LB-S and 24HB-S had lower F_v_/F_m_ values indicating stress occurred. What’s more, 24HB-S had a statistically lower F_v_/F_m_ value than 24LB-S indicating that having a higher blue light percentage during a static 24h photoperiod might cause more stress to a plants photon capture ability than a light treatment containing a proportionally lower blue fraction. Importantly, both 24h static light treatments (24LB-S and 24HB-S) had statistically lower F_v_/F_m_ values than all other light treatments including those under 24h dynamic light treatments (24LB-D and 24HB-D).

**Figure 2:**
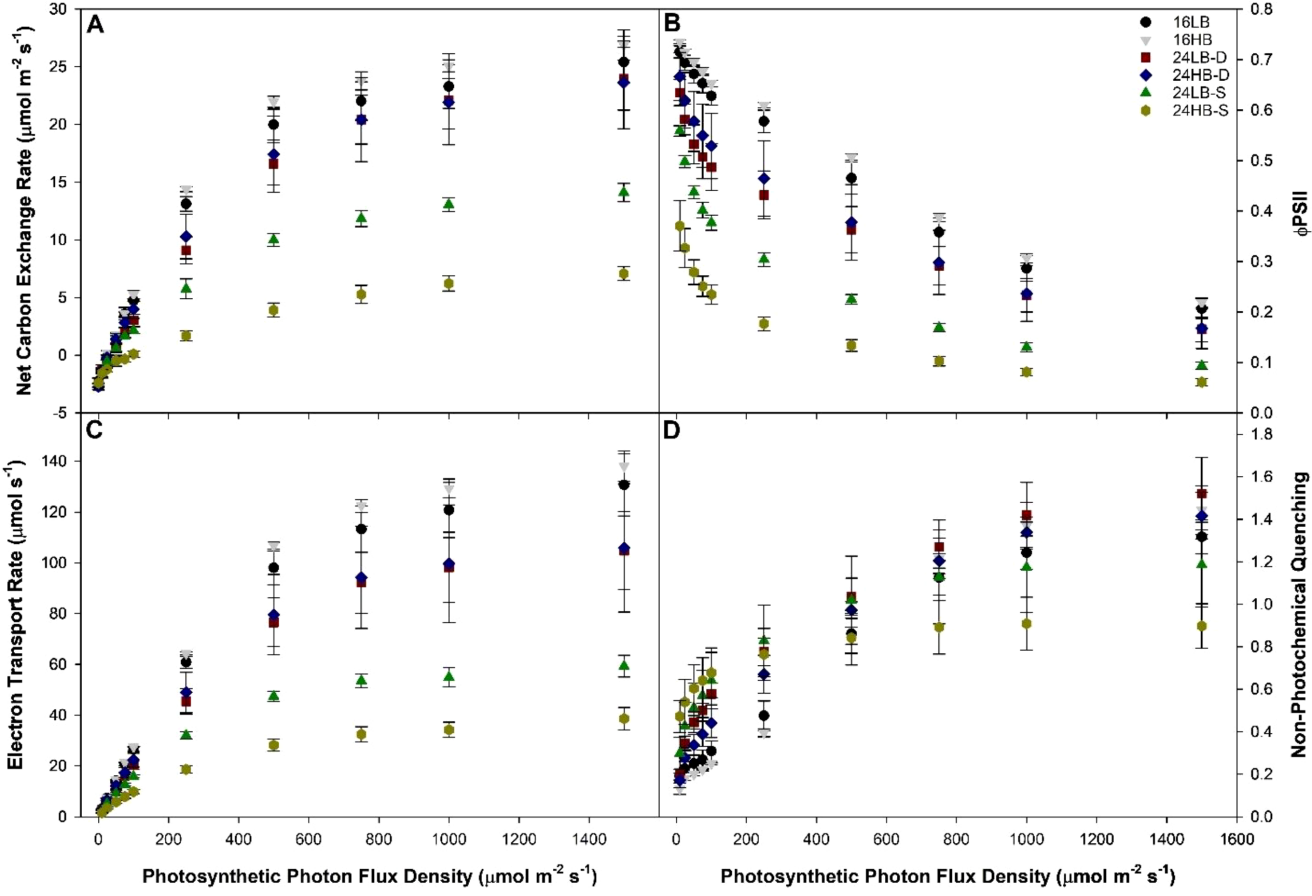
Light response curve **(A)**, the efficiency of photosystem II photochemistry [φPSII; **(B)**], electron transport rate [ETR; **(C)**], and non-photochemical quenching [NPQ; **(D)**] of cv. ‘Tomary’ leaves grown under various overhead supplemental lighting treatments at different photosynthetic photon flux densities (PPFD). Data points and error bars represent the average and standard error (n=3) of three randomly selected leaves from each treatment.

In contrast to dark-adapted chlorophyll fluorescence measurements which puts the plant in an artificially dark state, light-adapted chlorophyll fluorescence allow for the elucidation of the plant response under various real-world lighting conditions. In **Figure 2**, we present the response of ‘Tomary’ leaves to various PPFDs examining the net carbon exchange rate (**Figure 2A**), efficiency of PSII photochemistry (φPSII; **Figure 2B**), Electron transport rate (ETR; **Figure 2C**), and non-photochemical quenching (NPQ; **Figure 2D**). As the PPFD increased, the net carbon exchange rate of leaves under all light treatments increased as well (**Figure 2A**). Notably, leaves grown under the 24LB-S and 24HB-S treatments had reduced maximum photosynthetic rates compared to the other treatments (**Table 3**). The light compensation point (LCP) of 24HB-S was the highest among all light treatments. In addition, both static 24h light treatments (24LB-S and 24HB-S) had lower quantum yields compared to 16LB, 16HB, and 24HB-D indicative of a reduced ability to use incoming radiation for carbon fixation (**Table 3**).

**Table 3:** Summary of physiological parameters of cherry tomato cv ‘Tomary’ as determined with a Li-COR 6800 with a red/blue (90/10) light source.

Treatment	A_max_ (µmol m^-2^ s^-1^)	LCP (µmol m^-2^ s^-1^)	QY (µmol m^-2^ s^-1^/ µmol_photon_ m^-2^ s^-1^)	φPSII	ETR (µmol m^-2^ s^-1^)	NPQ
16LB	27.51 ± 2.29^A^	31.38 ± 1.63^B^	0.0737 ± 0.002^A^	0.21 ± 0.02^A^	130.7 ± 12.2^A^	1.32 ± 0.08^AB^
16HB	29.10 ± 1.55^A^	26.21 ± 2.86^B^	0.0733 ± 0.003^A^	0.22 ± 0.01^A^	138.1 ± 6.0^A^	1.44 ± 0.11^AB^
24LB-D	27.28 ± 2.78^A^	39.11 ± 5.98^B^	0.0522 ± 0.006^AB^	0.17 ± 0.02^A^	104.8 ± 15.4^A^	1.52 ± 0.17^A^
24HB-D	26.05 ± 4.21^A^	33.55 ± 3.40^B^	0.0644 ± 0.008^A^	0.17 ± 0.04^A^	105.9 ± 25.3^A^	1.42 ± 0.11^AB^
24LB-S	16.01 ± 0.97^B^	42.59 ± 7.32^B^	0.0453 ± 0.003^B^	0.09 ± 0.01^B^	59.3 ± 4.3^B^	1.19 ± 0.20^AB^
24HB-S	9.39 ± 0.61^C^	88.54 ± 13.88^A^	0.0223 ± 0.002^C^	0.06 ± 0.01^B^	38.6 ± 4.4^B^	0.90 ± 0.10^B^

**Figure 3:**
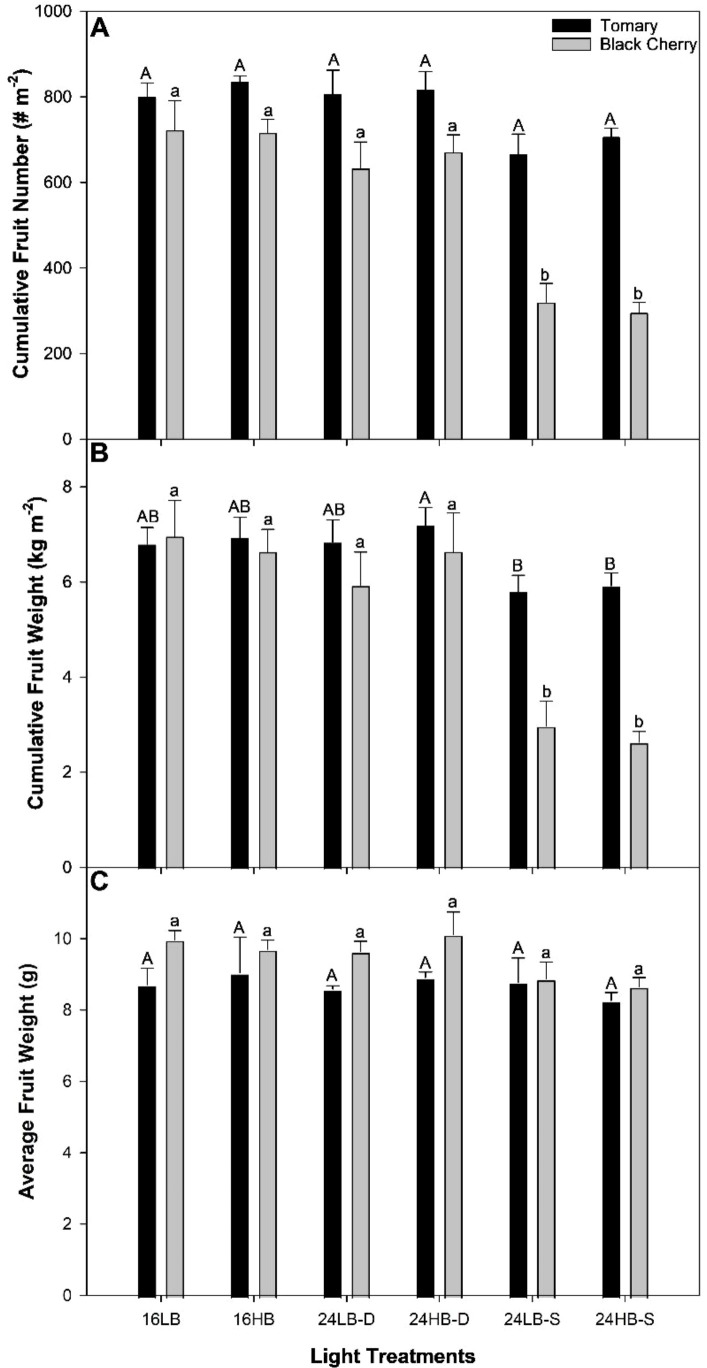
Total yield parameters from tomato cv. ‘Tomary’ (black bars) and ‘Black Cherry’ (grey bars) grown under all supplemental light treatments. Harvest began on January 24^th^, 2023 and culminated on April 17^th^, 2023. **(A)** represents the cumulative fruit number on an area basis, **(B)** represents the cumulative fruit weight on an area basis (yield), and **(C)** represents the average fruit weight. The bars and standard error bars represent the average and standard error of the mean of three independent replicates (n=3). Within each panel, different letter groups (A, B) represent a statistical difference as determine by a one-way ANOVA with a Tukey-Kramer adjustment (p<0.05). Upper case letters signify statistical differences for ‘Tomary’ and lower case letters for ‘Black Cherry’.

Measurements were made at a CO_2_ level of 1000 µL L^-1^, leaf temperature of 22^o^C, and a relative humidity of 60%. φPSII, ETR, and NPQ were determined at a PPFD of 1500 µmol m^-2^ s^-1^. Physiological parameters are calculated using the gas exchange and chlorophyll fluorescence curves found in **Figure 3**. Letter groups (A, B, C) represent significant difference between the lighting treatments at p<0.05 with a *post-hoc* Tukey-Kramer adjustment.

Light-adapted chlorophyll fluorescence measurements can give increased insight into the workings of the photosynthetic machinery. The efficiency of photosystem II photochemistry (φPSII) is an increasingly important metric which can assess the stress response of a leaf/plant through the understanding of light absorption and utilization. Both 24LB-S and 24HB-S had the lowest φPSII indicating that they were unable to utilize incoming radiation (**Table 3**). Additionally, in all light levels, except for 24LB-S at the 10 µmol m^-2^ s^-1^, both 24LB-S and 24HB-S had lower φPSII than leaves grown under other light treatments (**Figure 2**). This clearly illustrates 24 h static light treatments are detrimental to plant health and can lead to photoperiodic injury.

The electron transport rate (ETR) is a measurement akin to net carbon exchange rate. In this way, the results in **Figure 2C** showed very similar trends to that in **Figure 2A**, a rise in the ETR as PPFD increased with both 24LB-S and 24HB-S having the lowest values (**Table 3**). Non-photochemical quenching (NPQ) is a protective mechanism used by plants to dissipate excess light energy as heat. Both 24LB-S and 24HB-S had the highest NPQ values of all treatments when the PPFD was low (<500 µmol m^-2^ s^-1^; **Figure 2D**). However, an inflection point occurred around 500 µmol m^-2^ s^-1^ where above this PPFD, leaves from the 24LB-S and 24HB-S treatments began to have lower values than leaves grown under all other treatments. At 1500 µmol m-2 s-1, it was observed that 24LB-D had the highest value of NPQ and 24HB-S had the lowest (**Table 3**).

### Fruit production

Cumulative fruit number was not observed to be statistically different (p=0.0543) across all treatments in ‘Tomary’, although, both 24LB-S and 24HB-S had lower cumulative fruit numbers (**Figure 3A**). With respect to cumulative fruit weight on an area basis (yield), 24LB-S and 24HB-S had statistically the lowest yield while all 16 h and 24 h dynamic treatments had high yields in ‘Tomary’ (**Figure 3B**). Interestingly, both dynamic 24 h light treatments (24LB-D and 24HB-D) produced a similar number of fruit and cumulative fruit weight to 16LB and 16HB.

A drastic decrease in cumulative fruit number was observed from both 24LB-S and 24HB-S compared to other light treatments in ‘Black Cherry’ (**Figure 3A**). Subsequently, both treatments had statistically lower cumulative fruit yield than all other treatments (**Figure 3B**). Similar to ‘Tomary’, it was notable that 24LB-D and 24HB-D produced similar cumulative fruit number and.

yield to the 16 h controls. In addition, the average fruit weight was statistically similar among all light treatments in in both cultivars respectively (**Figure 3C**). The similarity between average fruit weight in each treatments correlated with a reduction in cumulative fruit number from the 24 h static treatments suggests that flower/fruit abortion was the main factor impacting yield in these treatments.

Regardless of photoperiod, the treatments with a high percentage of blue had an increase in electricity usage throughout the production period compared to the low blue treatments (**Table 4**). At the same blue light percentage, the electricity consumption was lowest in the 24h static treatments, likely due to the energy savings via dimming the fixtures.

**Table 4:** Electricity use and cost for all light treatments as determined using Sollum Technologies SUNaaS® platform and IESO (IESO, 2025) and OEB (OEB, 2022) pricing data respectively.

Treatment	Electricity use (kWh m^-2^)	Electricity cost ($ CAD m^-2^)	Electricity-use-efficiency (kWh kg^-1^ fruit weight)	Electricity-cost-efficiency ($ CAD kg^-1^ fruit weight)
‘Tomary’				
16LB	311	22.85	46.0 ± 2.4^A^	3.38 ± 0.18^AB^
16HB	354	26.02	51.4 ± 3.0^A^	3.78 ± 0.22^A^
24LB-D	310	19.85	45.8 ± 3.0^A^	2.93 ± 0.19^ABC^
24HB-D	347	22.54	48.5 ± 2.4^A^	3.15 ± 0.16^AB^
24LB-S	258	13.51	44.0 ± 3.4^A^	2.30 ± 0.18^C^
24HB-S	326	17.07	53.5 ± 3.1^A^	2.80 ± 0.16^BC^
‘Black cherry’				
16LB	311	22.85	45.6 ± 4.5^C^	3.35 ± 0.33^C^
16HB	354	26.02	53.9 ± 3.6^C^	3.96 ± 0.26^C^
24LB-D	310	19.85	54.0 ± 4.2^C^	3.45 ± 0.46^C^
24HB-D	347	22.54	53.6 ± 5.9^C^	3.48 ± 0.38^C^
24LB-S	258	13.51	93.9 ± 9.1^B^	4.92 ± 0.39^B^
24HB-S	326	17.07	126.5 ± 10.5^A^	6.62 ± 0.55^A^

Electricity-use-efficiency (EUE) and electricity-cost-efficiency (ECE) were calculated by dividing the respective totals for the production period by the total fruit weight. Letter groups (A, B, C) represent significant difference between the lighting treatments (n=3) at p<0.05 with a *post-hoc* Tukey-Kramer adjustment. Statistics were not completed on electricity use and electricity cost since they were the same for all treatments. Of note, values are higher than what would be expected for a commercial greenhouse due to the need for additional fixtures to maintain light uniformity within each treatment plot.

Electricity cost is a function of when the electricity was used during the 24 h period and the cost of electricity during the hour. Similar to electricity use, all treatments with a high blue percentage had a higher associated electricity cost compared to the low blue counterparts (**Table 4**). When normalized for blue light percentages, 24 h dynamic treatments had a lower electricity cost compared to the 16 h treatments. This is mainly due to the 24 h lighting treatments taking advantage of Ontario’s low nighttime electricity prices. The 24 h static treatments had the lowest overall costs due to lower monthly delivery charge (lower peak light intensity or peak electricity consumption during a month).

In ‘Tomary’, the electricity-use-efficiency (EUE; the amount of electricity used to produce a kg of fruit, the lower the better) was similar among all treatments (**Table 4**). In ‘Black Cherry’ both of the 24h static light treatments had highest EUEs than the other treatments with the 24HB-S treatment having the highest of all treatments. This indicates that the conversion of electricity to marketable biomass was the worst in the 24h static treatments.

The ECE provides information related to the expenditure associated with lighting with each treatment. In ‘Tomary’ 24LB-S had the lowest ECE meaning the electricity cost to produce a kilogram of tomatoes was the lowest (**Table 4**). In addition, 24HB-S had a lower ECE than 16HB. While both 24 h static treatments had the lowest yield, electricity cost had larger reduction than yield reduction. While the other treatments are statistically similar, in practical terms, both 24 h dynamic treatments have appreciable ECE improvement. For example, 24LB-D has a 45¢ decrease in electricity cost per kilogram of fruit produced. Similarly, 24HB-D has a 63¢ decrease compared to the 16HB treatment.

Although the electricity used was the same for ‘Black Cherry’ a much different story was observed. The ECE was similar between the 16 h and 24 h dynamic treatments (**Table 4**) while both 24 h static treatments were significantly higher. The difference between ‘Tomary’ and ‘Black Cherry’ is due to the larger yield reduction from both 24 h static treatments in the latter cultivar. Together, this data shows the importance of cultivar specific research even within the same species type (i.e., both cherry tomatoes).

**Figure 4:**
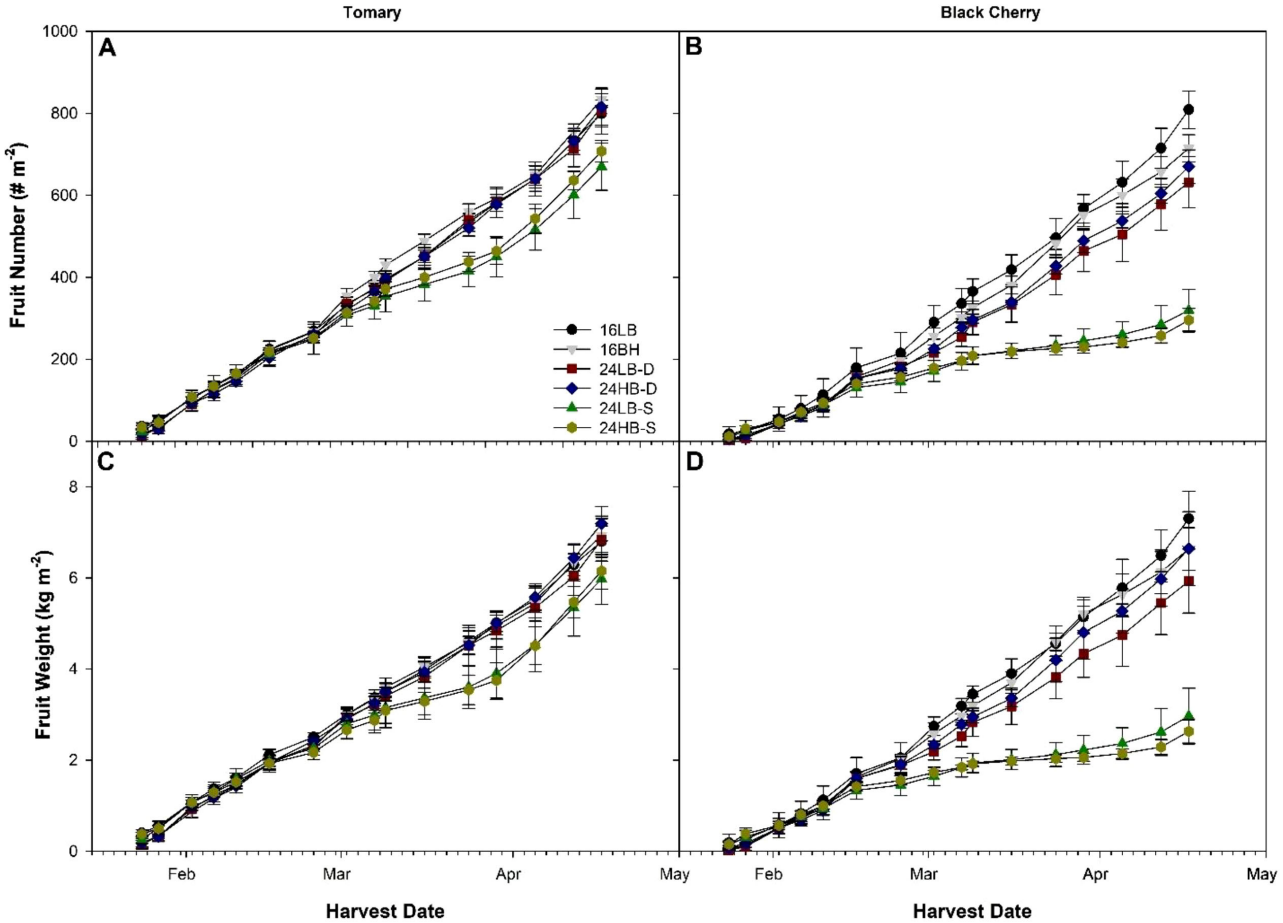
Cumulative fruit number **(A, B)** and cumulative fruit weight **(C, D)** of tomato cv. ‘Tomary’ **(A, C)** and ‘Black Cherry’ **(B, D)** grown under all overhead supplemental light treatments as recorded from January 24^th^, 2023 to April 17^th^, 2023.

The production patterns show interesting trends among the light treatments in both cultivars. In ‘Tomary’, both fruit number and fruit weight are similar among all 6 light treatments during the early period of production (January-March; **Figures 4A, C**). However, a deviation occurred at the beginning of March. During this time period we see that both 24LB-S and 24HB-S had reduced fruit number and fruit weight compared to the other light treatments. This trend continued for approximately a month. Interestingly, around the beginning of April, we observed a recovery in both fruit number and fruit weight from plants grown under 24LB-S and 24HB-S. By the end of the experiment, the individual harvest data among all the treatments were again similar. However, 24LB-S and 24HB-S did not fully make up the lost production observed during the month of March.

A similar trend was observed in ‘Black Cherry’ (**Figures 4B, C**). However, in contrast to the patterns observed in ‘Tomary’, the deviation in yield from plants under 24LB-S and 24HB-S occurred slightly earlier (mid-February) and was much more drastic – illustrated by the greater difference compared to the other treatments. The reduction in yield persisted for much longer in ‘Black Cherry’ and was still evident in early April. Only during the last harvest did we observe an inclination that yields may be increasing. Perhaps if the trial was continued, the individual harvest data for plants grown under 24LB-S and 24HB-S would start to mirror that of the other treatments.

### Fruit quality and antioxidant content

**Figure 5:**
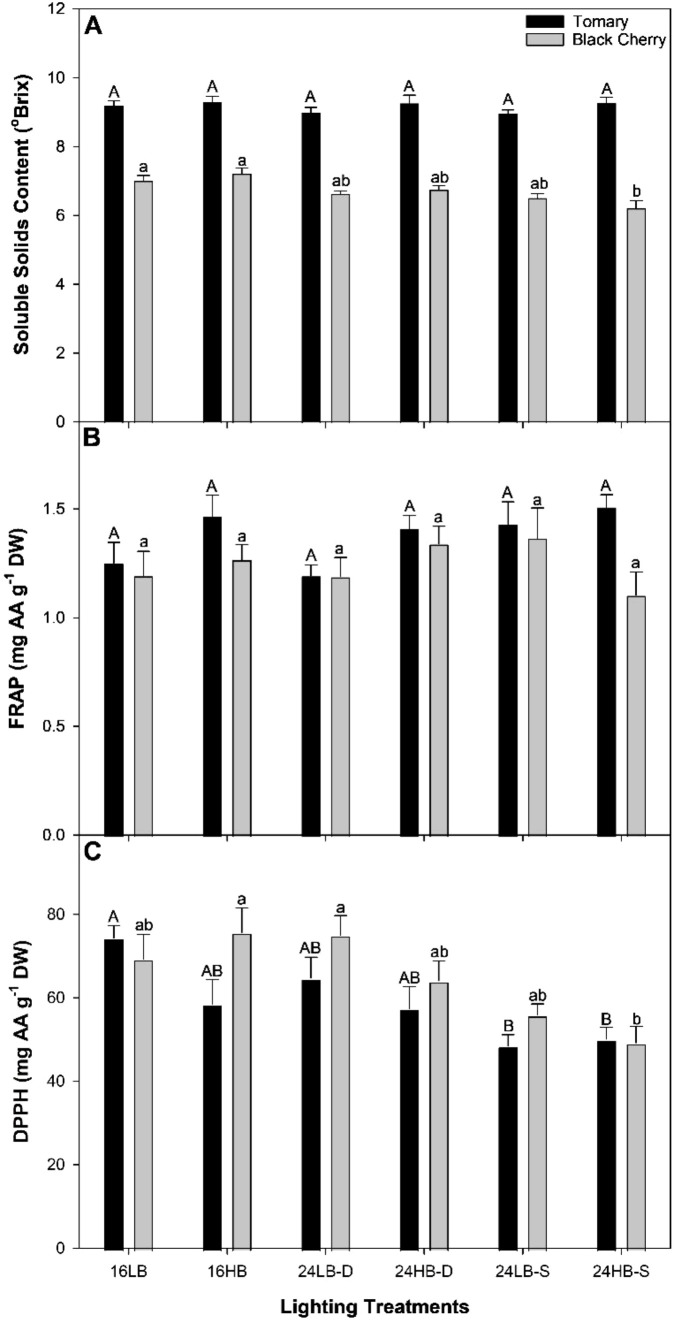
Soluble Solids Content [Brix; **(A)**] and antioxidant activities as measured by ferric reducing antioxidant power [FRAP; **(B)**] and 2, 2, -diphenyl-1-picrylhydrazyl [DPPH; **(C)**] in cv. ‘Tomary’ and ‘Black Cherry’ from fruit harvested on February 24^th^, 2023. The bars and standard error bars represent the average and standard error of the mean of six independent replicates (n=6). Within each panel, different letter groups (A, B) represent a statistical difference as determine by a one-way ANOVA with a Tukey-Kramer adjustment (p<0.05). Upper case letters signify statistical differences for ‘Tomary’ and lower case letters for ‘Black Cherry’.

**Figure 6:**
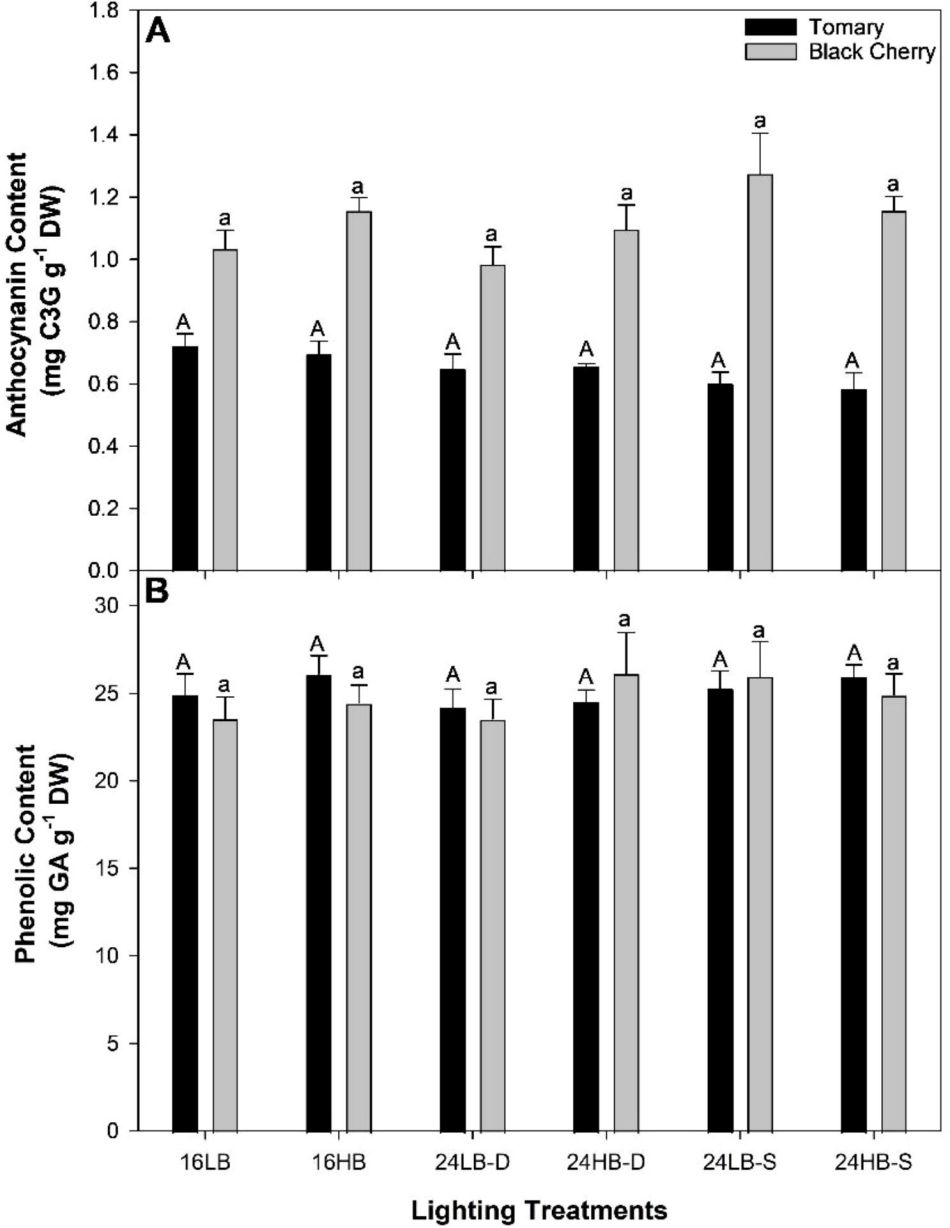
Anthocyanin **(A)** and Phenolic **(B)** content in cv. ‘Tomary’ and ‘Black Cherry’ from fruit harvested on February 24^th^, 2023. The bars and standard error bars represent the average and standard error of the mean of six independent replicates (n=6). Within each panel, different letter groups (A, B) represent a statistical difference as determine by a one-way ANOVA with a Tukey-Kramer adjustment (p<0.05). Upper case letters signify statistical differences for ‘Tomary’ and lower case letters for ‘Black Cherry’.

Fruit quality assessment measurements for both cultivars under all light treatments can be found in **Figures 5**, **6**. The soluble solids contents (SSC; also known as Brix) in ‘Tomary’ were similar between all light treatments (**Figure 5A**). However, in ‘Black Cherry’ both 16LB and 16HB had higher SSC than 24HB-S. Ferric reducing antioxidant power (FRAP) assay and 2, 2, -diphenyl-1-picrylhydrazyl (DPPH) assay measured the antioxidant content within the tomato fruits (**Figures 5B, C**). In ‘Tomary’ and ‘Black Cherry, the difference in FRAP was not statistically significant although it was always slightly higher in the lighting treatments with high proportion of blue light except for 24HB-S. DPPH was the lowest in both 24LB-S and 24HB-S while the highest in 16LB (**Figure 5C**) in ‘Tomary’. Similarly in ‘Black Cherry’, 24HB-S had the lowest DPPH values while 16HB and 24LB-D had the highest (**Figure 5C**). Although the anthocyanin was much higher in ‘Black Cherry’ fruit compared to ‘Tomary’, the lighting treatments had no impact on its content (**Figure 6A**). In addition, the phenolic content in both ‘Tomary’ and ‘Black Cherry’ were similar among all treatments (**Figure 6B**).

## Discussion

### Dynamic lighting can mitigate injury during 24 h lighting

A lot of literature suggest that regardless of the light spectrum a plant is grown under (i.e., white, red+blue, artificial solar), a constant spectrum for 24 h will result in leaf injury in vascular plants (Murage and Masuda, 1997; Demers and Gosselin, 1999; Haque et al., 2015; Velez-Ramirez et al., 2017; Pham et al., 2019). In this study, it is clear that the use of a dynamic lighting strategy implementing both a shift in light spectrum (white during the day to blue at night) and a decrease in PPFD had no negative impact on overall plant health. The often used F_v_/F_m_ metric to assess plant health were similar between the 16 h photoperiod treatments (16LB and 16HB) and the two 24 h dynamic treatments (24LB-D and 24HB-D) while the two 24 h static treatments (24LB-S and 24HB-S) had lower values, indicating injury. The difference in the evidence of photoperiodic injury (leaf chlorosis) was also visually apparent between the 24 h dynamic treatment and 24 h static treatment (**Supplementary Figure 2**). In addition, specifically in ‘Black Cherry’, plants grown under 24LB-S and 24HB-S had significantly lower yield indicating that the leaf injury accumulated into reduced fruit production.

Taken together, these results show that dynamic light treatments can mitigate photoperiodic injury in greenhouse tomatoes grown under 24 h supplemental lighting. However, the dynamic strategy used in this experiment had two components: 1) a reduction in PPFD from day to night and 2) a change in light spectrum from white to blue. Elucidating which of these two components are responsible for the lack of photoperiod injury can hint towards a mechanistic explanation.

In an indoor study when artificial light is the sole light source, Velez-Ramirez et al. (2017) found that 16 h red/blue mixture consisted of red (80 µmol m^-2^ s^-1^) and blue (20 µmol m^-2^ s^-1^) followed by 8 h of the red/blue mixture or red only (100 µmol m^-2^ s^-1^) or blue only (100 µmol m^-2^ s^-1^) at a total DLI 8.64 mol m^-2^ d^-1^ led to leaf injury (chlorosis and reduced F_v_/F_m_) in the CL sensitive tomato line A131 but not in the CL tolerant line (CLT). On the other hand 24 h artificial solar light resulted in leaf injury on both tomato lines. In our previous greenhouse research on TOV (tomato on the vine) cultivars, 16 h red/blue mixed light (185 µmol m^-2^ s^-1^ at 80% red and 20% blue) followed by 95 µmol m^-2^ s^-1^ red only or blue only light did not induce leaf injury but 24 h of 152 µmol m^-2^ s^-1^ white LED lighting resulted in significantly leaf injury and yield reduction even if the supplemental DLI was the same (13.1 mol m^-2^ d^-1^, Hao et al, 2023). In another greenhouse TOV tomato trial with both top lighting and inter-lighting at 14.4 mol m^-2^ d^-1^ DLI, a 24 h white top light of 115 µmol m^-2^ s^-1^ (at 52 µmol m^-2^ s^-1^ inter-lighting, the inter-lighting did not cause leaf injury) induced severe leaf injury (Hao et al., 2023, Greensys2023, Cancun, Mexico, oral presentation and abstract). In this study, the 24 h static light with high percentage of blue light (30%) resulted in more reduction in F_v_/F_m_ and photosynthesis than the one with lower percentage of blue light (10%) at the same DLI 14.4 mol m^-2^ d^-1^. Therefore, light spectrum does play a role in CL injury.

Both light intensity and spectrum affect the redox regulation of plant metabolism (Borbely et al., 2022). High DLIs increase photo-assimilates which can increase antioxidants or free-radical scavenging substances and thus improve plant tolerance to the ROS from CL. High percentage of blue light increases ROS production (Consentino et al., 2015; El-Esawi et al., 2017), which might have surpassed the free-radical scavenging capacity at the low DLI (14.4 mol m^-2^ d^-1^) in this study and thus resulted in the more severe CL injury in the 24HB-S light treatment (**Figure 5C**). Future study on CL impact on redox mechanism is needed to confirm this hypothesis. Comparing low blue to high blue treatments within their respective photoperiod, it is clear that the addition of blue light provided no advantage to plants with respect to CL mitigation. In addition, under the most stressful conditions (i.e., 24 h static lighting), a higher percentage of blue light conveyed more injury than a low percentage (**Figure 1**). This suggests that increasing the amount of blue light, at least while background radiation is present, hinders the plants ability to deal with CL stress. While it is likely that this is due to the increase ROS production under such lighting conditions (El-Esawi et al., 2017) further research is warranted.

Other environmental factors, such as temperature, can also modulate the endogenous circadian rhythm of a plant (De Leone and Yanovsky, 2024). Haque et al. (2017) provided evidence that the introduction of a thermoperiod (a 10 °C drop in temperature between the day and night) during CL eliminated injury compared to CL with constant temperature. Our current study involved a temperature reduction between the day and night, although, not as significant as the aforementioned study. It is important to note that although there was a thermoperiod present, our 24 h static light treatments still produced CL related injury (**Figure 1**). Two theories arise to explain the difference: 1) either the temperature difference in our study (~6°C) was not enough to regulate the circadian rhythm, or 2) light regulation enacts a stronger circadian rhythm response than temperature. Further experimentation regarding the interaction between light intensity, spectrum, timing, and temperature is needed in order to determine how to best regulate the environment during long photoperiod lighting.

### Practical implications of CL

The use of CL holds promising cost savings for growers while also having potential benefits with reduction of GHG emissions. Using a continuous photoperiod allows for the movement of electricity usage from the day to the night (i.e., peak shaving), a reduction in light intensity (lower monthly electricity delivery charge) to achieve the same DLI, and a reduction in fixture installation capacity when employed at scale. All of these factors can amount to appreciable savings for growers.

Here, we show that simply using a CL strategy can reduce the ECE by over $1 kg^-1^ (**Table 4**). Although, in this example (comparing 16LB to 24LB-S) a overall reduction in production was also observed. When production was maintained (16LB vs. 24LB-D), the reduction in ECE was observed to be 45¢ (**Table 4**). While not statistically significant, such a reduction in cost per kilogram of fruit amortized over many acres can have significant practical financial implications for growers. During our study alone, yields were approximately 7 kg m^-2^ in ‘Tomary’ (**Figure 3B**). The cost savings from the 24LB-D compared to the 16LB was $3.15 m^-2^. With approximately 4046 m^2^ in an acre, simply switching to the 24LB-D lighting strategy from the 16LB strategy would save growers approximately $12, 700 per acre per year in electricity costs – significant savings. However, it is important to note that our results also showed a cultivar dependent interaction; results for ‘Black Cherry’ under 24 h dynamic lighting with a low blue percentage did not reduce cost compared to the 16LB treatment. Therefore, it is important that growers take into account the cultivar they are using before implementing such a lighting strategy.

Additionally, the use of a low blue light containing light treatment had cost saving benefits compared to high blue light containing strategies in most cases (**Table 4**). Since the reduction of blue light was made up with red light (a higher efficacy wavelength), less electricity was used which amounted to a lowed ECE from low blue containing treatments.

Electricity use between the 16 h and 24 h dynamic treatments was very similar when accounting for the percentage of blue light (**Table 4**). Due to the dimming properties of the light fixtures, 24 h static treatments used less electricity than their 16 h and 24 h dynamic counterparts. Although electricity use was similar between 16 h and 24 h dynamic treatments, the 24 h treatments are able to take advantage of off-peak renewable electricity sources. In Ontario, Canada, this means preferentially utilizing hydroelectric and nuclear electricity generation methods – both low GHG emission sources (NREL, 2021; IESO, 2025). So, although the total amount of electricity is very similar, the 24 h lighting treatments have used electricity from low emission sources and thus result in lower overall GHG emissions.

### Plant recovery under increasing solar radiation

An interesting and unexpected phenomenon observed was the apparent recovery of plants exhibiting signs of photoperiodic injury later in the experiment. This recovery was noticed in the yield metrics for ‘Tomary’ and also hinted at in those of ‘Black Cherry’, although much later in the experiment (**Figure 4**) when solar radiation intensity and natural photoperiod (i.e., DLI) increased (**Supplementary Figure 3**). A similar result was also observed in our previous experiments in tomato where after plants showed photoperiodic injury under long photoperiods, and then recovered later in the growth cycle when solar radiation got stronger (Lanoue et al., 2021).

Photoperiodic injury in tomato is always first observed in the leaf. This is because the leaves are exposed to the CL condition early in their life cycle when they are at the top of the plant canopy. The realization of CL injury through yield is delayed until fruit developed weeks after it is observed on the leaf. Hence, the reduction in yield observed during March in ‘Tomary’ (**Figure 4C**) correlates with the injury observed in the upper leaves as assessed in February (**Figure 1**). This also means that the apparent recovery in yield observed in mid-April correlates with leaves being produced in early to mid-March. During this period, the natural solar radiation is increasing (**Supplementary Figure 3**). Additionally, in an effort to maintain similar DLIs between all lighting treatments, the supplemental lights were not turned off, regardless of natural light levels. This means, that during the spring months, plants are being exposed to an ever increasing DLI and higher daily PPFD while recovery is observed. This phenomenon has been observed multiple times during our various CL experiments (Lanoue et al., 2019; Hao et al., 2023, Hao et al., 2025). Therefore, we designed and conducted a greenhouse study (Hao et al., 2025) on CL and DLI interaction, which demonstrated high DLI increase tomato’s CL tolerance. During periods of high solar radiation and high DLI, plants are able to cope better with long photoperiods. It has been proposed that photorespiration plays a role in CL tolerance. Cucumbers, being a CL tolerant plant, have a higher photorespiratory rate than tomatoes (Marie et al., 2024). Interestingly, photorespiration is known to increase during exposure to high light (Zhang et al., 2024). High photorespiration may provide more intermediates which may be used to produce free-radical scavenging substances and thus increase the tolerance to CL.

Photoperiodic injury is likely a cause of circadian asynchronicity where the exogenous environment and endogenous plant rhythms do not align (Velez-Ramirez et al., 2017). During periods of low natural sunlight such as that in December-February where CL injury was observed, the difference between daytime maximum PPFD and the nighttime PPFD in 24LB-S and 24HB-S is minimal. However, in the spring months, the highest daytime PPFD could reach 1000 µmol m^-2^ s^-1^ with the supplemental and solar radiation combined. Given the nighttime PPFD was only 168 µmol m^-2^ s^-1^, this nearly 850 µmol m^-2^ s^-1^ swing could reset the circadian synchronicity between the exogenous environment and plants’ own circadian rhythm. This may be at least partially the case for our DLI x CL interaction trial when the high DLI light treatment with much larger day and night light intensity difference eliminated the harmful effects of CL (Hao et al., 2025). Furthermore, high day and night temperature difference can improve tomato tolerance to CL (Haque et al., 2017). In the spring months, the actual day-night temperature difference is much larger than during December-February due to high day temperature from strong solar radiation which could also reset the circadian synchronicity and allowed the plants to recover.

## Conclusion

Our data confirms that the use of a dynamic 24 h lighting strategy which involved both a change in light intensity and spectrum between the day and night is an efficient method to mitigate photoperiodic injury during CL. In this study, we were able to move 15% of total DLI from the day period (6:00-22:00) to the night period (22:00-6:00). In Ontario, Canada and other regions of the world which use time-of-use electricity pricing, this also means that there was a cost-savings for the growers while maintaining yield – however this was cultivar dependent. Our data also showed that growing cherry tomato plants under 24 h dynamic lighting does not reduce the fruit quality while growing under some 24 h static lighting treatments can not only hinder yield but also fruit quality. Additionally, we found that high percentage of blue light did not improve tomato’s tolerance to CL injury and also did not improve fruit nutritional quality nor yield. Therefore, light recipes with low percentage of blue light are more energy-efficient for cherry tomatoes grown under 24 h dynamic lighting.

## Data Availability

The original contributions presented in the study are included in the article/**Supplementary Material**. Further inquiries can be directed to the corresponding author.
