# Supplementary material for: Dynamic lighting mitigates photoperiodic injury in greenhouse tomatoes
Source: Front Plant Sci. 2026 Feb 4;17:1731972. doi: 10.3389/fpls.2026.1731972 (PMC12913140; doi:10.3389/fpls.2026.1731972)
Supplement: Supplementary file 1 [file Supplementaryfile1.docx]

**SUPPLEMENTARY FIGURES**


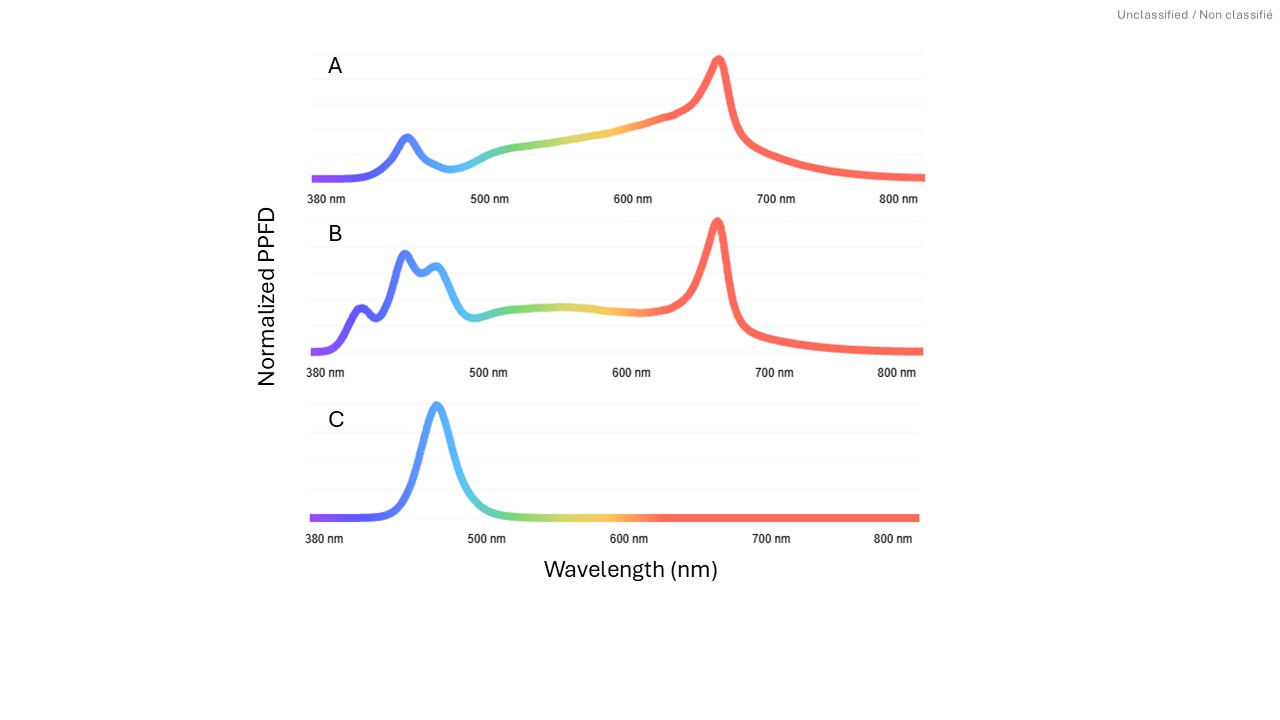
**Supplementary Figure 1:** Normalized PPFD spectral outputs of the LB (A), HB (B), and blue night (C).

**
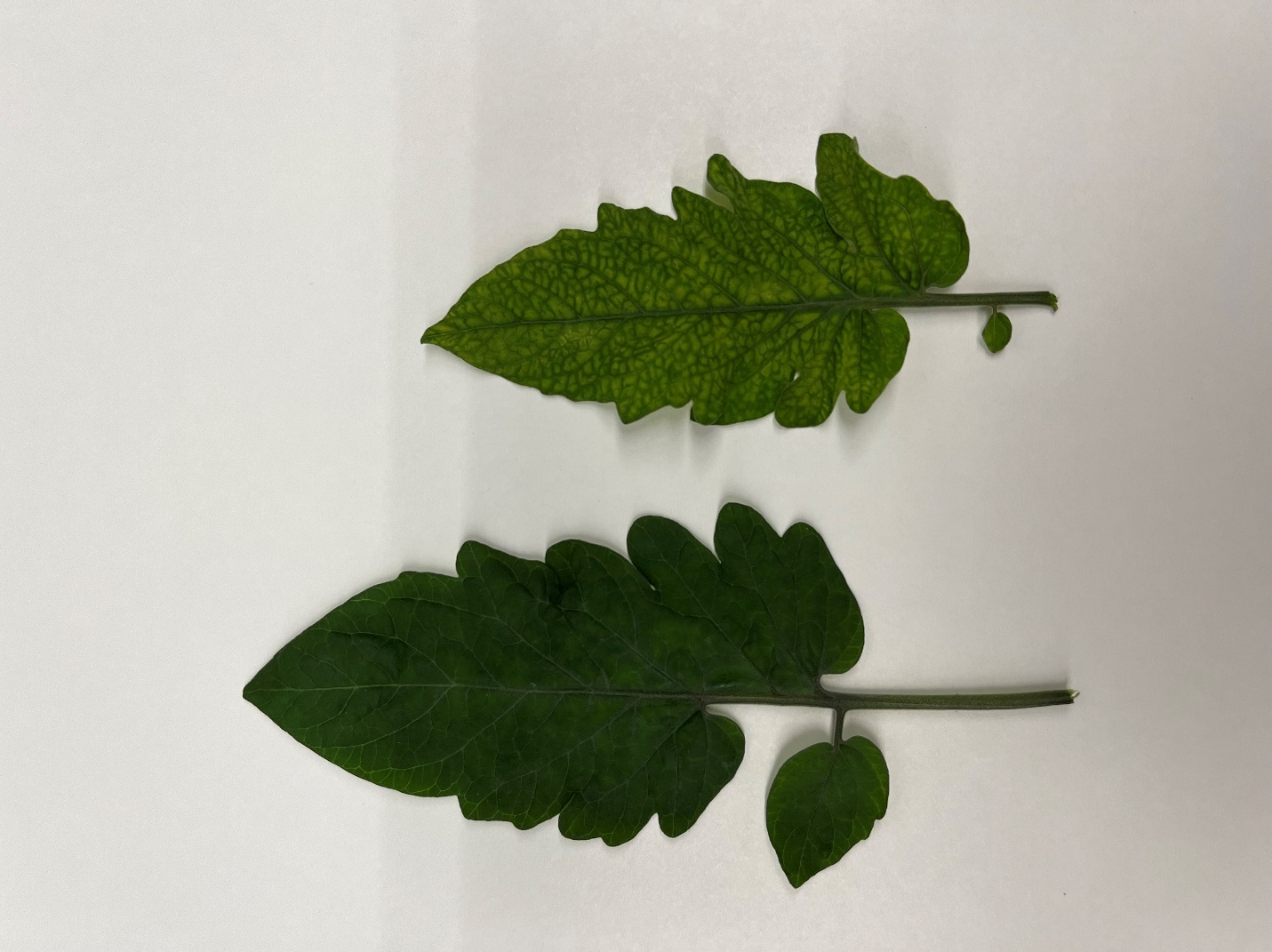
**

**Supplementary Figure 2:** Leaflets from a plant under a 24 h dynamic light treatment (left) and a 24 h static light treatment (right). The leaflet from the static light treatment on the right shows clear signs of intervenial chlorosis indicative of CL injury. Photo taken on February 8^th^, 2023.


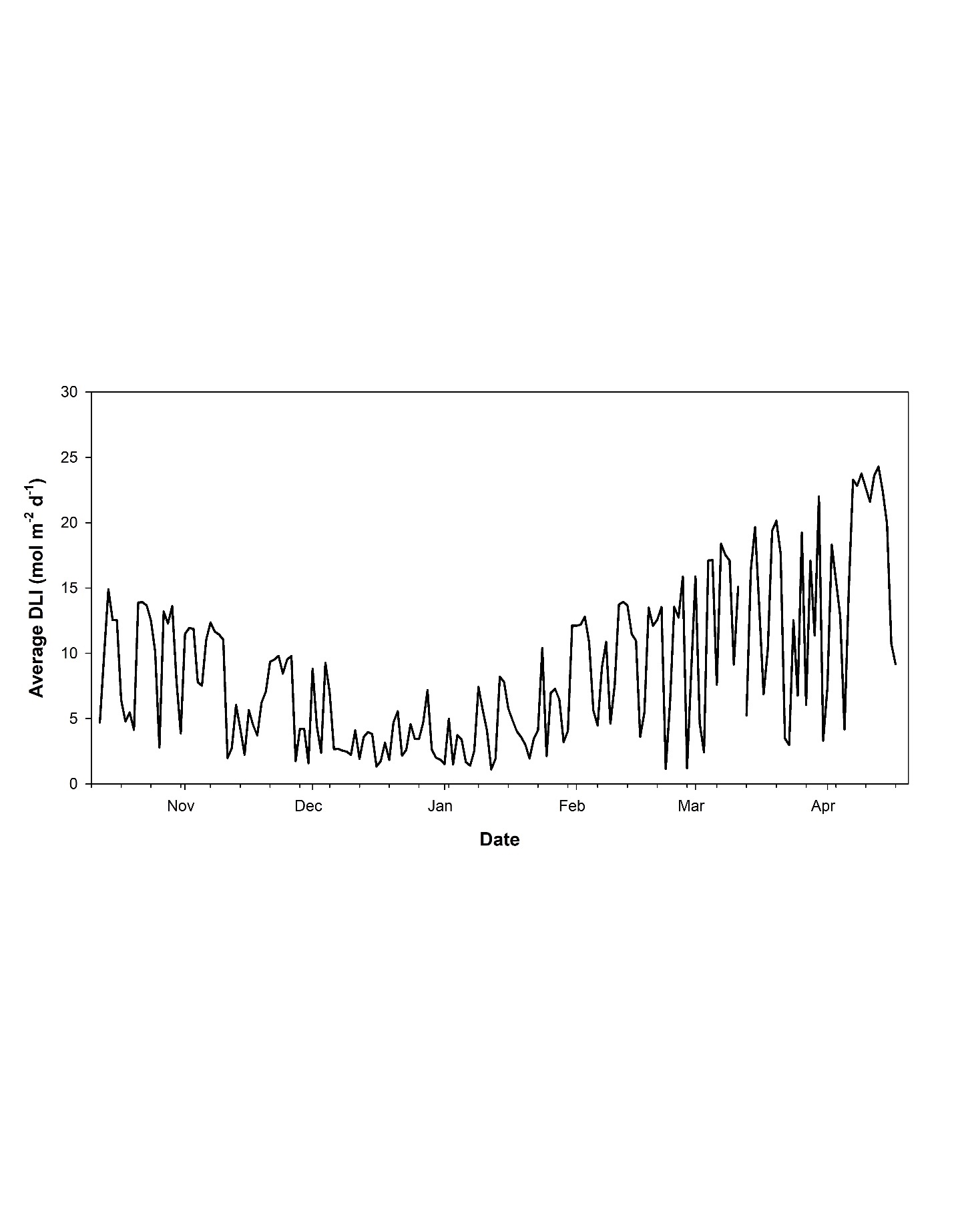
**Supplementary Figure 3:** Average DLI throughout the experiment as measured using a Li-COR LI-200R pyranometer converted form W m^-2^ to µmol m^-2^ s^-1^ using a conversion factor of 2.1 to convert from 400-1100nm to PAR. Readings were taken above the greenhouse and then corrected for an approximate 50% transmissivity to account for shading from the greenhouse structure, lighting fixtures, and shade curtains. Breaks in the line indicate periods of time which were not recorded due to a technical malfunction.
